# Reporting of race in genome and exome sequencing studies of cancer: a scoping review of the literature

**DOI:** 10.1038/s41436-019-0558-2

**Published:** 2019-06-04

**Authors:** Adrienne Nugent, Kelly R. Conatser, Llaran L. Turner, James T. Nugent, Esther May B. Sarino, Luisel J. Ricks-Santi

**Affiliations:** 1 Cancer Research Center, Hampton University, Hampton, VA USA; 2 Graduate Programs in Public Health, University of New England, Portland, ME USA; 3 Department of Pediatrics, Joint Base Langley-Eustis, Hampton, VA USA; 4 Department of Pediatrics, F. Edward Hebert School of Medicine, Uniformed Services University of the Health Sciences, Bethesda, MD USA; 5 Edward E. Brickell Medical Sciences Library, Eastern Virginia Medical School, Norfolk, VA USA

**Keywords:** genome sequencing, exome sequencing, race, cancer, disparities

## Abstract

**Purpose:**

Minorities are often underrepresented in clinical cancer research yet the frequency of reporting of race in genomic sequencing studies of cancer is unknown. This scoping review determines the rate at which race is reported as a demographic variable, the factors associated with reporting of race, and the participation rates of minority populations.

**Methods:**

PubMed was systematically searched from 1 January 2010 through 15 November 2018 and 11,014 studies were assessed for eligibility. Publications reporting genome or exome sequencing data for patients with one of the ten most common cancers in the United States were included.

**Results:**

A total of 231 publications containing sequencing data from 15,721 unique patients met inclusion criteria. Race was reported in 37% of studies compared with 84% of studies reporting age and 85% reporting gender. Reporting of race was associated with cohort size, sequencing method, familial cancer, cancers with disparities, and reporting of age and gender. Minority populations were significantly underpowered to detect recurrent pathogenic variants in most cancers.

**Conclusion:**

Race is underreported as a demographic variable in genomic sequencing studies of cancer. Substantially increased efforts are needed to sequence patients from underrepresented populations to reduce health disparities in patients of non-European ancestry.

## INTRODUCTION

Genome sequencing (GS) and exome sequencing (ES) have transformed the clinical ability to identify pathogenic variants in cancer. As these next-generation sequencing (NGS) technologies have become more cost-effective and ubiquitous, the rate of genetic sequencing data in the literature has increased exponentially.

One aspect frequently overlooked in cancer NGS studies is the racial composition of the patient cohort.^1,2^ The cancer burden in the United States disproportionately affects minorities due to numerous factors including access to care, socioeconomic status, and genetics. The impact of ancestry-related genetic variation on cancer incidence and mortality disparities in minority populations has been documented for breast, lung, prostate, colorectal, melanoma, and kidney cancer.^3,4^ The National Institutes of Health (NIH), the Cancer Moonshot Initiative, and major cancer research organizations advocate for diversity in clinical cancer research,^5^ yet minority populations are frequently underrepresented in clinical trials^6,7^ and genome-wide association studies.^8,9^ Despite the abundance of clinical sequencing studies performed in the past decade, the accrual of minorities and reporting of race in NGS studies of cancer remain unknown.

We have performed a scoping review of the literature to comprehensively describe the representation of minority populations in publications containing GS/ES sequencing data for the ten most common cancers in the United States. We chose to perform a scoping review to identify the available evidence, clarify concepts, and identify gaps in knowledge concerning race reporting and minority inclusion in NGS cancer studies that are contributing to disparities in clinical cancer research. Scoping reviews aim to identify characteristics of studies to examine how research is conducted at an overview level but do not assess risk of bias as in a systematic review.^10^ The objectives of this scoping review were to measure the frequency of race reporting as a demographic variable in NGS cancer studies, quantify the rate of minority participation in these study cohorts, and explore factors associated with race reporting.

## MATERIALS AND METHODS

This review was conducted using the Preferred Reporting Items for Systematic reviews and Meta-Analyses extension for Scoping Reviews (PRISMA-ScR).^11^

### Search strategy

PubMed was systematically searched for studies published between 1 January 2010 and 15 November 2018 using search strategies designed by an experienced medical science librarian (E.M.B.S.) and stated in the Supplemental Appendix. Additional sources were identified through screening of The Cancer Genome Atlas (TCGA) publications and related studies.

### Study selection

The studies included in this review performed GS or ES on tissue from patients diagnosed with one of the ten most common cancers in the United States at sufficient depth to identify rare somatic or germline variants. Low-pass GS studies were excluded. All studies had senior authors from a US institution and a clinical cohort that included at least one patient from the US for which sequencing data had not previously been reported. Studies were required to perform GS or ES on patient tissue. A total of 399 publications performing only targeted gene panel sequencing were excluded. Sequencing data from cell lines, xenografts, single cell, circulating tumor DNA, and nonhuman subjects were excluded. Case reports, review articles, meeting abstracts, pan-cancer, and multicancer studies were also excluded. A.N. reviewed studies for inclusion in November 2018 and consulted with J.T.N. and L.J.R.S. to resolve uncertainties. The study selection process was performed according to the PRISMA-ScR guidelines and additional details are provided in the Supplemental Appendix.

### Data extraction

Data abstraction was performed independently by two of four investigators (A.N., K.R.C., L.L.T., J.T.N.) for each publication. Discrepancies were resolved through communication between the reviewers. The extracted data included relevant bibliographic details (study title, first author, year of publication, journal, and journal impact factor), demographic characteristics of the patient population (race, ethnicity, age, and gender), and study details (cancer type, cohort size, sequencing technique, NIH funding, data availability, and associated clinical study). Race and ethnicity were defined by NIH notice NOT-OD-15-089 and encompassed five racial categories: White, Black or African American, Asian, American Indian or Alaska Native (AI/AN), or Native Hawaiian or Other Pacific Islander (PI) and two ethnic categories: (1) Hispanic or Latino or (2) non-Hispanic or non-Latino. TCGA and Therapeutically Applicable Research to Generate Effective Treatments (TARGET) studies were considered to include race, ethnicity, age, and gender even when not explicitly stated, as these data are available in online databases referenced in the publications. For the quantification of minority populations, patient data were retrieved from included publications and the Genomic Data Commons (GDC) Data Portal accessed on 18 January 2019. Although not all patients in the GDC Data Portal were from publications included in this review, these patients were included in the quantitative analysis because their data are publicly available to researchers.

### Statistical analyses

Statistical analyses were performed using StataSE 12.0 software (StataCorp LP, College Station, TX). Variables not specifically stated in the study were coded as missing. Individual study characteristics were compared between studies reporting or not reporting race using the Chi-square test for proportions and two-sided Mann–Whitney U test for medians. Logistic regression was performed to estimate adjusted odds ratios and 95% confidence intervals for the association between race reporting and study-level characteristics. Power calculations were performed using the www.tumorportal.org power calculator and previously defined cancer variant frequencies.^12,13^

## RESULTS

### Search results

A total of 11,014 studies were assessed for eligibility and 10,615 articles were excluded based on review of the title and abstract. An additional 168 full text articles were excluded for reasons shown in the Supplemental Appendix. In all, 198 studies passed screening and were included in this review, in addition to 33 studies identified from other sources, totaling 231 publications.

### Study characteristics

The 231 included publications contained sequencing data from 15,721 unique patients. Study characteristics are summarized in Table 1 and detailed descriptions are provided in Table S1. A mean of 23 studies were retrieved for each cancer type (range 11–32), 17 publications were from TCGA, 3 were from TARGET, and 41 reported data from patients enrolled in clinical studies. A total of 52 studies reported both GS/ES and targeted gene panel sequencing data, of which only GS/ES data were included in this analysis.

**Table 1:** Characteristics of genome and exome sequencing studies

Variable	Race reported (*n* = 85)	Race not reported (*n* = 146)	*P* value^a^	OR (95% CI)^b^	*P* value^c^
Gender reported—no. (%)	83 (98%)	114 (78%)	<0.001	10.53 (1.97–56.19)	0.006
Age reported—no. (%)	80 (94%)	115 (79%)	0.002	1.95 (0.59–6.45)	0.27
Familial disease—no. (%)	17 (20%)	9 (6%)	0.001	3.71 (1.43–9.63)	0.007
Cohort size—median (IQR)	31 (12–112)	20.5 (7–48)	0.007	1.00 (1.00–1.01)	0.01
Known disparity—no. (%)	70 (82%)	95 (65%)	0.005	2.26 (1.07–4.75)	0.03
GS included—no. (%)	25 (29%)	24 (16%)	0.02	3.96 (1.69–9.28)	0.002
Clinical study—no. (%)	10 (12%)	31 (21%)	0.07	0.48 (0.19–1.23)	0.13
Journal impact factor—median (IQR)	10.3 (5.2–27.1)	12.4 (8.0–27.1)	0.21	0.98 (0.96–1.01)	0.15
NIH funded—no. (%)	50 (59%)	74 (51%)	0.23	1.18 (0.61–2.29)	0.62
Publication year—median (IQR)	2015 (2014–2017)	2016 (2014–2017)	0.49	1.05 (0.87–1.28)	0.59
Available data—no. (%)	44 (52%)	82 (56%)	0.52	0.84 (0.43–1.64)	0.61

*CI*  confidence interval, *GS*  genome sequencing, *IQR* interquartile range, *NIH* National Institutes of Health, *OR* odds ratio.

^a^*P* values are based on Pearson’s Chi-square test for categorical variables and the Mann–Whitney U test for continuous variables.

^b,c^Adjusted odds ratios and *P* values were estimated with use of a multivariate logistic regression model with reporting of race as the dependent variable. Values were adjusted for gender reporting, age reporting, familial versus sporadic disease, cohort size, cancers with known ancestral genetic disparities, inclusion of GS, patient enrollment in clinical studies, journal impact factor, NIH funding, publication year, and data availability.

### Reporting of demographic variables

**Fig. 1:**
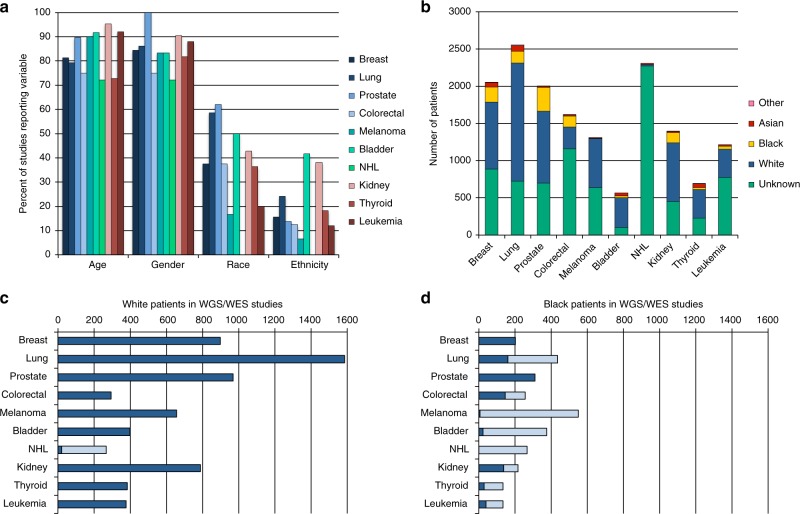
**Reporting of race in genome/exome sequencing (GS/ES) studies.** **a** The percent of studies reporting age, gender, race, and ethnicity as a demographic variable. **b** The number of patients included in all studies as identified by race. **c**,**d** The number of **c** White and **d** Black patients with published sequencing data. The number of patients with existing data is shown in dark blue and the number of additional patients needed to sequence to reach 90% power to detect a pathogenic variant present in 10% of patients is shown in light blue. *NHL* non-Hodgkin lymphoma.

Few studies reported race (*n* = 85, 37%) or ethnicity (*n* = 39, 17%) while the majority of studies reported gender (*n* = 197, 85%) and age (*n* = 195, 84%). Reporting of race varied by cancer type, with the highest percentage of studies reporting race in prostate cancer (18/29, 62%) and the fewest in non-Hodgkin lymphoma (NHL) (0/18, 0%) (Fig. 1a).

### Factors affecting reporting of race

Publications reporting race were more likely to be familial studies (*P* = 0.001), have larger patient cohorts (*P* = 0.007), report gender (*P* < 0.001), report age (*P* = 0.002), and include GS (*P* = 0.02) (Table 1). Race was more likely to be reported in studies of cancers with a known ancestry-related genetic disparity (breast, lung, prostate, colorectal, kidney cancer, and melanoma) compared with those without (bladder cancer, NHL, thyroid cancer, and leukemia) (*P* = 0.005). NIH funding, journal impact factor, publication year, depositing of sequencing data in publicly available databases, and enrollment of patients in a clinical study were not significantly associated with reporting of race. In a multivariate logistic regression model, studies of familial cancers, studies of cancers with known genetic disparities, cohort size, inclusion of GS, and reporting of gender remained significantly associated with reporting of race (Table 1).

### Analysis of race in publications

Race and ethnicity were rarely discussed in the publications. Of 85 studies that reported race, 36 (42%) analyzed or commented on the role of race in the context of their findings. Only 18/85 (21%) publications included a description of how race was determined, with 9 studies using self-reported race, 2 using physician-reported race, and 7 performing ancestry analysis by single-nucleotide polymorphism array or ancestry-informative markers (AIMs).

### Inclusion of minorities in sequencing studies

Race was provided for patients in study publications or the GDC Data Portal for 7790 of 15,721 (50%) patients (Fig. 1b). A total of 5042 (65%) patients for whom race was reported were from the 20 TCGA and TARGET studies included in this analysis. Of 85 publications that reported race, 24 (28%) reported patients from only one race (18 White, 6 Black). Black and Asian/PI patients comprised a greater percentage of sequencing study participants compared with the proportion of Black and Asian/PI incident cancer patients for 6/10 and 7/10 cancers, respectively (Supplemental Appendix). AI/AN populations were sequenced at lesser rates than incident cancer patients for all cancer types.

### Power to detect pathogenic variants by race

Of patients with race reported, 6373 (82%) were White, 1064 (14%) were Black, 316 (4%) were Asian/PI, 15 (0.2%) were AI/AN, and 22 (0.3%) were Other, similar to previous findings.^2,8^ The number of patients needed to sequence to achieve 90% power to detect a recurrent pathogenic variant present in at least 10% of patients was determined based on previously identified somatic pathogenic variant frequencies of individual cancers.^12,13^ The total number of published genomes and exomes from Whites exceeded this minimum threshold for 9 of 10 cancer types (Fig. 1c). However, in Blacks, only breast and prostate cancer had a sufficient number of cases to achieve this power (Fig. 1d). Asian/PI and AI/AN populations did not achieve this power for any cancer type.

## DISCUSSION

This scoping review and systematic analysis of genome and exome sequencing studies of the ten most common cancers in the United States found that race was significantly underreported as a demographic variable compared with age and gender. Previous analyses quantifying the inclusion of minorities in cancer NGS have been limited to TCGA^2^ and 23 single-race studies in the Database of Genotypes and Phenotypes.^8^ Here, we found that Black and Asian/PI, but not AI/AN, patients were included in sequencing studies at higher rates than incident patients for the majority of cancer types. However, the total number of minority patients with sequencing data remains significantly underpowered to detect pathogenic variants in all minority populations.

As the patient populations represented in research studies directly inform clinical decision-making and outcomes, substantially increased efforts are needed to sequence patients from minority populations to reduce health information disparities in patients of non-European ancestry. A more complete understanding of ancestral genetics has already yielded positive outcomes, such as beginning to explain why African American women have more aggressive triple negative breast cancer than Caucasian women,^14^ why African Americans with renal cell carcinoma are less likely to respond to treatment,^15^ and why children with acute lymphoblastic leukemia and >10% Native American ancestry are more likely to relapse.^16^ On the other hand, ancestry bias in clinical databases has resulted in genomic testing that is less informative and more costly in non-European patients^17^ and the failure to properly control for variants in minority populations has led to false positives and inaccurate conclusions of the genetic causes of cancer.^18,19^

One limitation of this scoping review is that the included publications are restricted to GS/ES and therefore other types of massively parallel sequencing are not considered. In addition, some publications include international cohorts with patients from other countries in addition to US patients. Finally, the ten cancers included in this study do not capture the full variation of cancer types and disparities in the United States.

A major benefit from systematically identifying these studies is that the opportunity exists to retroactively determine the ancestry of individual patients using AIMs. AIMs are more accurate than self-reported race and enable fine-scale resolution of admixture.^20^ Reanalysis of patients in these studies and inclusion of AIMs in future studies will enable deeper understanding of the contribution of ancestral genetics to identify population-specific subgroups, prognoses, drug responses, and treatment. Full characterization of these molecular subgroups will inform clinical decision-making and reduce racial disparities in cancer.

The role of ancestry-related genetic variation is an important yet understudied component of cancer genomic sequencing studies. Increasing minority participation and reporting in sequencing studies will help to define ancestry-related differences in the cancer genetic landscape to reduce the biological basis of racial disparities in cancer and improve clinical precision oncology in all patients.

## Supplementary information

Supplementary Appendix

Supplementary TableS1

## References

[1] Hindorff LA, Bonham VL, Ohno-Machado L (2018). Enhancing diversity to reduce health information disparities and build an evidence base for genomic medicine. Pers Med.

[2] Spratt DE (2016). Racial/ethnic disparities in genomic sequencing. JAMA Oncol.

[3] Ozdemir BC, Dotto GP (2017). Racial differences in cancer susceptibility and survival: more than the color of the skin?. Trends Cancer.

[4] Yuan J (2018). Integrated analysis of genetic ancestry and genomic alterations across cancers. Cancer Cell.

[5] Polite BN (2017). Charting the future of cancer health disparities research: a position statement from the American Association for Cancer Research, the American Cancer Society, the American Society of Clinical Oncology, and the National Cancer Institute. J Clin Oncol.

[6] Murthy VH, Krumholz HM, Gross CP (2004). Participation in cancer clinical trials: race-, sex-, and age-based disparities. JAMA.

[7] Chen MS (2014). Twenty years post-NIH Revitalization Act: enhancing minority participation in clinical trials (EMPaCT): laying the groundwork for improving minority clinical trial accrual. Cancer.

[8] Landry LG (2018). Lack of diversity in genomic databases is a barrier to translating precision medicine research into practice. Health Aff (Millwood).

[9] Sirugo G, Williams SM, Tishkoff SA (2019). The missing diversity in human genetic studies. Cell.

[10] Munn Z (2018). Systematic review or scoping review? Guidance for authors when choosing between a systematic or scoping review approach. BMC Med Res Methodol.

[11] Tricco AC (2018). Prisma extension for scoping reviews (PRISMA-ScR): checklist and explanation. Ann Intern Med.

[12] Lawrence MS (2014). Discovery and saturation analysis of cancer genes across 21 tumour types. Nature.

[13] Agrawal N (2014). Integrated genomic characterization of papillary thyroid carcinoma. Cell.

[14] Keenan T (2015). Comparison of the genomic landscape between primary breast cancer in African American versus white women and the association of racial differences with tumor recurrence. J Clin Oncol.

[15] Krishnan B (2016). Intrinsic genomic differences between African American and white patients with clear cell renal cell carcinoma. JAMA Oncol.

[16] Yang JJ (2011). Ancestry and pharmacogenomics of relapse in acute lymphoblastic leukemia. Nat Genet.

[17] Kessler MD (2016). Challenges and disparities in the application of personalized genomic medicine to populations with African ancestry. Nat Commun.

[18] Gerhard GS (2017). Pitfalls of exome sequencing: a case study of the attribution of HABP2rs7080536 in familial non-medullary thyroid cancer. NPJ Genom Med.

[19] Garofalo A (2016). The impact of tumor profiling approaches and genomic data strategies for cancer precision medicine. Genome Med.

[20] Mersha TB, Abebe T (2015). Self-reported race/ethnicity in the age of genomic research: its potential impact on understanding health disparities. Hum Genomics.

