# Supplementary material for: Reporting of race in genome and exome sequencing studies of cancer: a scoping review of the literature
Source: Genet Med. 2019 Jun 4;21(12):2676–80. doi: 10.1038/s41436-019-0558-2 (PMC6891161; doi:10.1038/s41436-019-0558-2)
Supplement: Supplementary file 1 — Supplementary Appendix [file 41436_2019_558_MOESM1_ESM.docx]

**Supplementary Appendix**

**Study selection**

The following search string was used to identify studies from PubMed:

(((((("The Cancer Genome Atlas"[TIAB] OR TCGA[TIAB] OR "breast neoplasms"[MH] OR "breast cancer"[TIAB] OR "breast carcinoma in situ"[MH] OR "carcinoma, ductal, breast"[MH] OR "breast neoplasms, male"[MH] OR "carcinoma, lobular"[MH] OR "hereditary breast and ovarian cancer syndrome"[MH] OR "inflammatory breast neoplasms"[MH] OR "triple negative breast neoplasms"[MH] OR "unilateral breast neoplasms"[MH] OR "prostatic neoplasms"[MH] OR "prostate cancer"[TIAB] OR "prostatic neoplasms, castration-resistant"[MH] OR "lung neoplasms"[MH] OR "bronchial neoplasms"[MH] OR "multiple pulmonary nodules"[MH] OR "pancoast syndrome"[MH] OR "pulmonary blastoma"[MH] OR "pulmonary sclerosing hemangioma"[MH] OR "lung cancer"[TIAB] OR "pulmonary cancer"[TIAB] OR "colorectal neoplasms"[MH] OR "colorectal cancer"[TIAB] OR "rectal cancer"[TIAB] OR "rectum cancer"[TIAB] OR "urinary bladder neoplasms"[MH] OR "bladder cancer"[TIAB] OR "urinary bladder cancer"[TIAB] OR "lymphoma, non-hodgkin"[MH] OR "non-hodgkins lymphoma"[TIAB] OR "thyroid neoplasms"[MH] OR "thyroid cancer"[TIAB] OR "thyroid carcinoma"[TIAB] OR "leukemia"[MH] OR leukeumia[TIAB] OR "blood cancer"[TIAB] OR "leucocythemia"[TIAB] OR "leukemia, myeloid"[MH] OR "leukemia, myeloid, acute"[MH] OR "leukemia, eosinophilic, acute"[MH] OR "leukemia, erythroblastic, acute"[MH] OR "leukemia, mast-cell"[MH] OR "leukemia, megakaryoblastic, acute"[MH] OR "leukemia, monocytic, acute"[MH] OR "leukemia, promyelocytic, acute"[MH] OR "melanoma"[MH] OR melanoma[TIAB] OR "hutchinson's melanotic freckle"[MH] OR "melanoma, amelanotic"[MH] OR "melanoma, experimental"[MH] OR "kidney neoplasms"[MH] OR "carcinoma, renal cell"[MH] OR "renal cell carcinoma"[TIAB] OR "nephroma, mesoblastic"[MH] OR "wilms tumor"[MH] OR "wilms tumor"[TIAB] OR "kidney cancer"[TIAB] OR "renal cancer"[TIAB] OR "renal pelvic cancer"[TIAB] OR "ureter cancer"[TIAB]))) AND (("whole genome sequencing"[MH] OR WGS[TIAB] OR "whole genome sequencing"[TIAB] OR "whole exome sequencing"[MH] OR WES[TIAB] OR "whole exome sequencing"[TIAB] OR "sequence analysis, DNA"[MH] OR "high-throughput nucleotide sequencing"[MH] OR "sequence analysis"[MH] OR "DNA mutational analysis"[MH] OR "sequence analysis, DNA"[MH] OR "sequence analysis, RNA"[MH] OR "genomics"[MH] OR "exome sequencing"[TIAB] OR "tumor sequencing"[TIAB] OR "deep sequencing"[TIAB] OR "massively parallel sequencing"[TIAB] OR "genomic sequencing"[TIAB] OR "genome sequencing"[TIAB] OR "genomic sequence"[TIAB]))) AND (("mutation"[MH] OR mutation[TIAB] OR "genetic predisposition to disease"[MH] OR "genetic predisposition*"[TIAB] OR "germ-line mutation"[MH] OR "germline variants"[TIAB] OR "exome"[MH] OR "whole exome"[TIAB] OR "whole genome"[TIAB] OR "genome, human"[MH])) AND (("2010/01/01"[PDat] : "2018/12/31"[PDat]))))

**Study selection process**

Articles were selected for inclusion based on a literature search and study selection process following PRISMA guidelines [1].

*^a^* Non-Hodgkin Lymphoma comprises Diffuse Large B-Cell Lymphoma and Follicular Lymphoma

*^b^* Leukemia comprises Acute Myeloid Leukemia and Chronic Lymphocytic Leukemia

**Extended study selection details**

The 10 most common cancers in the U.S. were defined by the Surveillance, Epidemiology, and End Results Program (SEER) and include breast, lung and bronchus, prostate, colon and rectum, melanomas of the skin, bladder, non-Hodgkin lymphoma (NHL), kidney and renal pelvis, thyroid, and leukemia [2]. As more than 60 NHL subtypes have been recognized by the World Health Organization, only the two most common forms of NHL in the U.S., Diffuse Large B-Cell Lymphoma and Follicular Lymphoma, were included in this analysis as they comprise approximately 30% and 20% of NHL cases, respectively, with no other subtype affecting more than 10% of cases. Similarly, only the two most common forms of leukemia (Acute Myeloid Leukemia and Chronic Lymphocytic Leukemia) were included.

Publications were considered to have patients enrolled in clinical studies if the publication contained information regarding an observational study or clinical trial with research study information available at www.clinicaltrials.gov. Publications were considered to have NIH funding if the research studies were NIH-coordinated or intramural projects or if a corresponding author stated funding from a research grant (R series) or career development award (K series) from the NIH.

U.S. cancer incidence rate data were retrieved from www.statecancerprofiles.cancer.gov and based on data from the *United States Cancer Statistics* from the Center for Disease Control and Prevention and the National Program of Cancer Registries [3]. The latest 5-year average annual count for all ages in the U.S. was used for calculations. For consistency with SEER statistics, cancer incident rates for Asians and Native Hawaiian or Other Pacific Islanders were combined [2]. The proportion of incident cancer patients was calculated for each cancer and race by dividing the average annual count for each race by the average annual count of the four races combined (White, Black, Asian/PI, AI/AN; all races included Hispanics). Similarly, the proportion of patients with sequencing data was determined for each cancer and race as a fraction of the total patients from the four races. These data are shown in Supplemental Table 2.

| **Supplemental Table 2.** Average annual cancer incidence (2011-2015) and genomic sequencing study participant percentages by race. Breast cancer incidence rates are for female breast cancer only. Cancer/race combinations for which the proportion of sequenced patients is greater than the incident cancer proportion are shown in bold. | | | | | | | | | | |
| --- | --- | --- | --- | --- | --- | --- | --- | --- | --- | --- |
|  |  |  |  |  |  |  |  |  |  |  |
| Average annual cancer incidence: proportion by race | | | | |  |  |  |  |  |  |
|  | Breast | Lung | Prostate | Colorectal | Melanoma | Bladder | NHL | Kidney | Thyroid | Leukemia |
| White | 83.8% | 86.2% | 80.8% | 83.1% | 98.9% | 92.2% | 87.9% | 84.7% | 85.2% | 88.1% |
| Black | 11.7% | 10.6% | 16.5% | 12.4% | 0.5% | 5.7% | 8.3% | 12.1% | 8.1% | 8.6% |
| Asian/PI | 3.9% | 2.5% | 2.2% | 3.7% | 0.3% | 1.8% | 3.3% | 2.3% | 6.1% | 2.8% |
| AI/AN | 0.6% | 0.6% | 0.4% | 0.7% | 0.2% | 0.3% | 0.5% | 0.9% | 0.6% | 0.6% |
|  |  |  |  |  |  |  |  |  |  |  |
| Genomic sequencing studies of cancer: proportion by race | | | | | |  |  |  |  |  |
|  | Breast | Lung | Prostate | Colorectal | Melanoma | Bladder | NHL | Kidney | Thyroid | Leukemia |
| White | 77.1% | **87.0%** | 74.3% | 64.8% | 97.3% | 85.4% | 54.1% | 83.7% | 82.6% | 86.9% |
| Black | **17.4%** | 8.8% | **24.4%** | **32.3%** | **0.9%** | 5.1% | 0.0% | **14.5%** | 6.2% | **9.2%** |
| Asian/PI | **5.3%** | **3.9%** | 1.2% | 2.6% | **1.8%** | **9.4%** | **45.9%** | 1.6% | **11.0%** | **3.5%** |
| AI/AN | 0.2% | 0.3% | 0.1% | 0.2% | 0.0% | 0.0% | 0.0% | 0.2% | 0.2% | 0.5% |

**SUPPLEMENTAL REFERENCES**

1. Moher, D., et al., *Preferred reporting items for systematic reviews and meta-analyses: The prisma statement.* Annals of Internal Medicine, 2009. **151**(4): p. 264-269.

2. Noone AM, H.N., Krapcho M, Miller D, Brest A, Yu M, Ruhl J, Tatalovich Z, Mariotto A, Lewis DR, Chen HS, Feuer EJ, Cronin KA (eds). *SEER Cancer Statistics Review, 1975-2015*. [cited <https://seer.cancer.gov/csr/1975_2015/>, based on November 2017 SEER data submission, posted to the SEER web site, April 2018.

3. *U.S. Cancer Statistics Working Group. U.S. Cancer Statistics Data Visualizations Tool, based on November 2017 submission data (1999-2015): U.S. Department of Health and Human Services, Centers for Disease Control and Prevention and National Cancer Institute;* <http://www.cdc.gov/cancer/dataviz>*, June 2018.*
